# The Sol–Gel Process, a Green Method Used to Obtain Hybrid Materials Containing Phosphorus and Zirconium

**DOI:** 10.3390/gels10100656

**Published:** 2024-10-13

**Authors:** Petru Merghes, Gheorghe Ilia, Bianca Maranescu, Narcis Varan, Vasile Simulescu

**Affiliations:** 1 “King Mihai I” University of Life Sciences from Timisoara, Calea Aradului 119, 300645 Timisoara, Romania; petrumerghes@usvt.ro (P.M.); narcisvaran@usvt.ro (N.V.); 2 Faculty of Chemistry, Biology, Geography, West University Timisoara, 16 Pestalozzi Street, 300115 Timisoara, Romania; gheorghe.ilia@e-uvt.ro

**Keywords:** sol–gel, phosphorus, zirconium, organic–inorganic hybrids, solid foam, surfactants

## Abstract

The sol–gel process is a green method used in the last few decades to synthesize new organic–inorganic phosphorus-containing hybrid materials. The sol–gel synthesis is a green method because it takes place in mild conditions, mostly by using water or alcohol as solvents, at room temperature. Therefore, the sol–gel method is, among others, a promising route for obtaining metal-phosphonate networks. In addition to phosphorus, the obtained hybrid materials could also contain titanium, zirconium, boron, and other elements, which influence their properties. The sol–gel process has two steps: first, the sol formation, and second, the transition to the gel phase. In other words, the sol–gel process converts the precursors into a colloidal solution (sol), followed by obtaining a network (gel). By using the sol–gel method, different organic moieties could be introduced into an inorganic matrix, resulting in organic–inorganic hybrid structures (sometimes they are also referred as organic–inorganic copolymers).

## 1. Introduction

One of the most important challenges for researchers involved in chemical synthesis nowadays is to reduce the negative effects of different processes on the environment. During the last decades, several green syntheses were developed to decrease the impact on the environment, by reducing the use and/or the production of pollutants and using solvents with low toxicity, in mild conditions. One of those methods is the sol–gel process, which will be described further. The sol–gel method represents a green technique of great interest widely used for synthesizing hybrid materials containing phosphorus, titanium, and/or zirconium. Of course, the sol–gel process could be used in different fields and other elements could even be involved, such as aluminum, silver, vanadium, tin, iron, boron, and so on, but the aim of the present review is the use of the sol–gel method for obtaining hybrids containing phosphorus and zirconium. There are several reasons to consider the sol–gel method as a green synthetic route. First of all, this method involves green solvents and uses mild conditions, such as low temperatures. In general, it is usually performed at room temperature, and the most used solvents are water and ethanol. Sometimes, alcohols with long hydrocarbon chains could also be used, due to their surfactant properties, such as hexanol, heptanol, octanol, decanol, dodecanol, and so on. Such alcohols behave as non-ionic surfactants; but, in aqueous solutions at a certain concentration, they will phase separate instead to form micelles.

Therefore, the sol–gel method is, among others, a promising route involved in the synthesis of metal phosphonates, as metal–organic frameworks (MOFs). The sol–gel method is a green chemistry alternative for obtaining such organic–inorganic phosphorus containing hybrids.

## 2. Sol–Gel Method in Phosphorus Chemistry

### 2.1. Gels and Gel-like Hybrid Materials

The sol–gel process takes place in two steps: first, the sol formation and further to this, the transition to the gel phase. The sol is a colloidal suspension of solid particles in a liquid, and on the other hand, the gel phase is a gelatinous network represented by the dispersion of a liquid in a solid material. In other words, the sol–gel process represents the conversion of the used precursors (with relatively low molar mass) into a colloidal system (sol), which is the precursor for obtaining a network (gel). By using the sol–gel method, it is possible to introduce a large variety of organic moieties bearing ionogenic groups into an inorganic matrix. Organic–inorganic hybrid materials containing different functional groups (alcoholic, carboxylic, sulphonic, etc.) have already been obtained by using the sol–gel method [1,2,3,4]. These hybrid materials showed, in general, very high thermal stability. The presence of the structural hydroxyl protons [2,3,4] indicates a good potential for these materials to exhibit solid-state proton conduction [1,5,6,7]. In addition to their high thermal stability, the proton conduction represents an important property required for different applications of great interest of such hybrid materials, in the fields of renewable energy. Therefore, discovering new proton conductors is an area of great interest nowadays due to the potential use of those compounds for water electrolysis units, sensors, or other electrochemical devices. By using the sol–gel process, several hybrid compounds could be obtained as organic–inorganic networks, including metal–organic frameworks (MOFs), heterocyclic and macrocyclic compounds, grafted materials, organic–inorganic copolymers containing phosphorus and different metal atoms as previously mentioned, and so on [1,8,9,10,11,12,13,14,15,16,17,18,19,20,21,22,23,24,25]. The obtained products could be used for promising applications also in the fields of catalysis, separation, and even medicine. The applications in medicine are mainly related to the strong mechanical properties of such hybrid materials, required for obtaining prostheses to replace compact bone tissues. For that purpose, the most important are the hybrid materials containing zirconium or titanium. First, titanium was mostly used, due to its strong mechanical resistance. But, in the last decades, zirconium actually started to replace titanium for different applications in this field.

Therefore, due to their structures, the obtained hybrids form organic–inorganic metal networks and also supramolecular structures. In addition to phosphorus, zirconium, titanium, or even boron, such hybrid materials also contain carbon from the organic moiety in their molecular structure. Practically, the P-O-M (where M is the used metal) bridges represent the only possibility in the sol–gel process to include the organic moieties on the hybrid structure if the precursors contain an organic part (for instance a phenyl group or an alkyl radical). This organic part will not react directly to the sol–gel process, due to the mild conditions involved, and as a consequence, it will be found also on the structure of the product, which will represent an organic–inorganic macromolecular structure, also containing carbon in addition to phosphorus, zirconium, or other elements. If the precursor contains the radical phenyl for example, the synthesized organic–inorganic hybrid will also contain the phenyl group. Otherwise, if the precursors would not contain any organic moiety, only inorganic compounds would be obtained.

**Figure 1:**
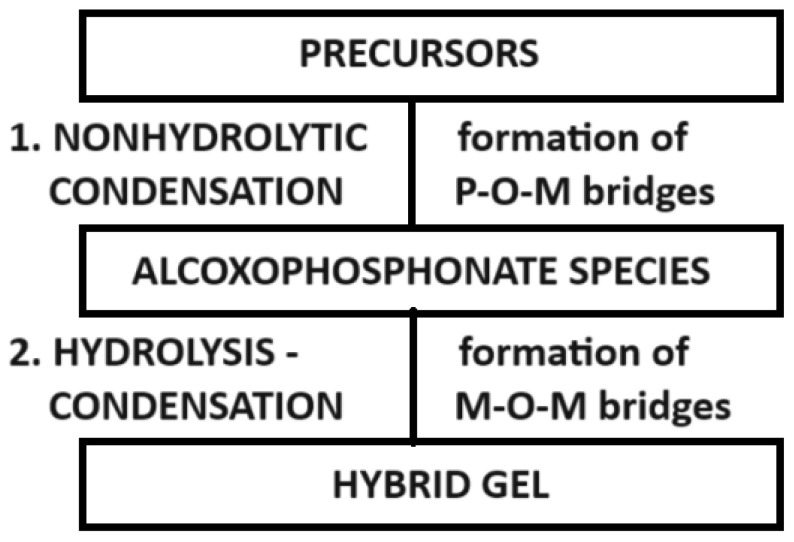
The nonhydrolytic step and the hydrolytic step take place during the sol–gel process.

As already mentioned, the only possibility to obtain the organic–inorganic structure is actually to obtain P-O-M bridges formed in the first step (the nonhydrolytic condensation). In addition to this, on the structure of the hybrids, M-O-M bridges also appear, but in the second step of the sol–gel process (the hydrolysis—condensation step, Figure 1).

All of these bridges obtained between the heteroatoms contained in the organic–inorganic hybrid compound form a complex structure that could be regarded as macromolecular, characterized by high mass and also by the so-called gyration radius as in the case of proteins or other organic polymers. From the dependence of gyration radius vs. molar mass, for a polymer, the conformation could be obtained. Unfortunately, it is not the case for those organic–inorganic hybrids, because their structure could not be easily predicted, as for organic polymers/copolymers, with one or two monomer units that are constantly repeated. For a better understanding, a comparison with organic copolymers is necessary. For instance, the example of alginates that are copolymers containing glucuronic acid (G unit) and mannuronic acid (M unit) as monomer units could be considered. Alginates of different sources could contain a different order of those units, which are repeated in their structure with high molar mass. The order is not necessary consecutively (i.e., M-G-M-G-M-G and so on). Sometimes, the alginates could contain a different order and a different ratio of the repeating units of the monomers from its structure, mannuronic acid and glucuronic acid, respectively. This was observed also in the structures of organic–inorganic hybrid copolymers synthesized by the sol–gel method. In most cases, function of the molar ratio of the used precursors, the ratio and the order of P-O-Zr and Zr-O-Zr bridges from the structure of the obtained hybrids could be changed. The determination of the conformation, in the case of biopolymers, is possible by using SEC-MALLS techniques. This method (SEC-MALLS) represents an absolute technique and it is applicable only for soluble compounds, but MALLS alone could also be used for not soluble materials (it could analyze solid materials).

Hybrid materials with a rigid inorganic backbone and the flexible organic part containing at least one P-C bond (either aliphatic or aromatic, as described), with groups and functionalities that bear protons, were already synthesized by the sol–gel process [7]. Therefore, the sol–gel method is a promising route for obtaining several classes of compounds of great interest nowadays, to be used and involved in the mentioned applications. The main advantage of the sol–gel method is that it represents a green alternative that could replace some of the classical syntheses to reduce the impact on the environment.

### 2.2. Organic–Inorganic Hybrid Networks

The sol–gel method, also so-called “Chimie Douce”, takes place in mild conditions, mostly at room temperature, and by using green solvents. The sol–gel syntheses are performed usually in water or in alcohol. It is a modern process used in the last decades for obtaining several organic–inorganic hybrid materials containing different functional groups, for instance, alcoholic, carboxylic, or sulphonic groups [2,3,4]. Organic–inorganic hybrid materials that have a rigid inorganic backbone and flexible organic groups with functionalities that bear protons (-OH, -COOH, -SO_3_H) are of especially great interest because their number of surface protons could be modified and controlled, mainly by changing the synthesis parameters, such as temperature, reaction time, molar ratio, and the precursors. Due to the surface protons and due to their complex structures, as organic–inorganic networks, these new hybrid materials are generally expected to have solid-state proton conduction [1,5,6,7].

By using the sol–gel process, several hybrid compounds could be obtained, such as heterocyclic structures and organic–inorganic hybrid networks with promising applications as already mentioned [24,25,26,27,28,29,30,31,32,33,34,35,36,37,38,39,40,41,42,43,44,45,46,47,48] in the field of energy, separation, catalysis, and even in medicine, as follows:

– Photocatalysis [29];
– Catalysis of aerobic oxidation by a vanadium phosphonate hybrid material [26];
– Catalysis for the synthesis of benzimidazoles [45];
– Catalysis for the hydrogenation of ketones [40];
– Dye removal materials from wastewater [36];
– Membranes with ion exchange properties [5,6,28,35];
– Energy and electrochemical applications [33], including hybrid materials with incorporated dyes [15] for dye-sensitized solar cells (DSSC);
– Adsorption and separation [29,31,46];
– Synthesis of biocompatible materials for medicine [27].

All of these potential applications increased the interest in such organic–inorganic hybrids in the last decade. The applications in medicine are mainly related to biocompatible materials with strong mechanical properties, used to replace different tissues. Also, some phosphonic and phosphinic acids organic–inorganic hybrids derivatives were studied from the point of view of their activity against different human osteosarcoma cells [27]. Future research should be directed also toward elucidating the mechanisms of cellular interactions of those derivatives, also considering long-term effects and potential clinical applications.

Osteosarcoma is one of the most severe malignant pathologies that affect the bone system and accounts for over 3 million cases per year, worldwide. The organophosphorus compounds have been recently investigated for their anti-osteosarcoma activity, with good anti-proliferative effects. The mechanism involved is the inhibition of a specific protease (neutral endopeptidase) responsible for carcinogenesis. The optimum concentration was found to be in the range between 2.5 and 5 mM for an exposure of the cells for 24 h. [27]. When used as a precursor for obtaining MOFs, 2-carboxyethylphenylphosphinic acid showed a better influence and activity, in comparison with phenyl phosphonic acid and phenyl phosphinic acid. These findings showed a favorable therapeutic index, but further investigation is needed, before their use as therapeutics, in preclinical evaluations.

The hybrid materials with different incorporated dyes [15], also synthesized by using the sol–gel process, could be used for obtaining dye-sensitized solar cells (DSSC). This is possible because these materials show good porosity, which indicates facile diffusion of redox mediators within the obtained layer. The porosity is required for the application in DSSC to obtain a better interaction with a surface-bound dye. The incorporated dye practically represents the sensitizer.

**Figure 2:**
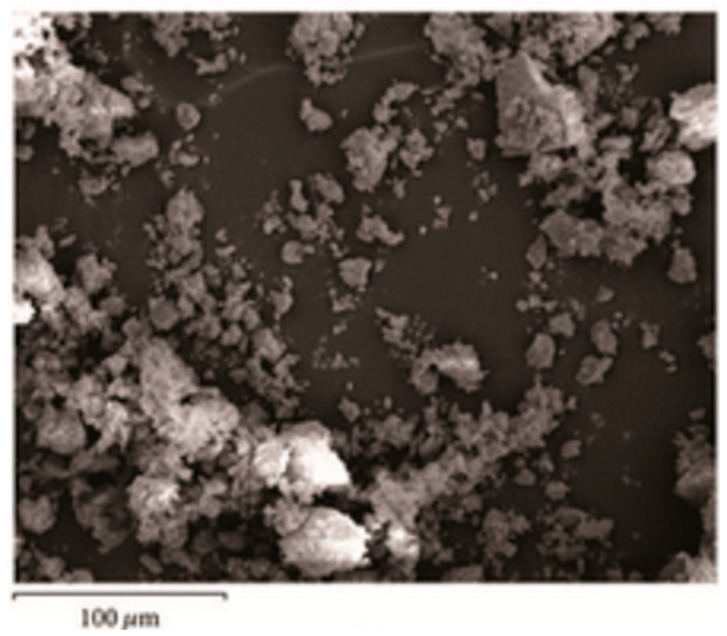
SEM image of an organic–inorganic hybrid synthesized by the sol–gel method, at a molar ratio of 2:1 (excess of phenyl phosphonic acid) [15].

**Figure 3:**
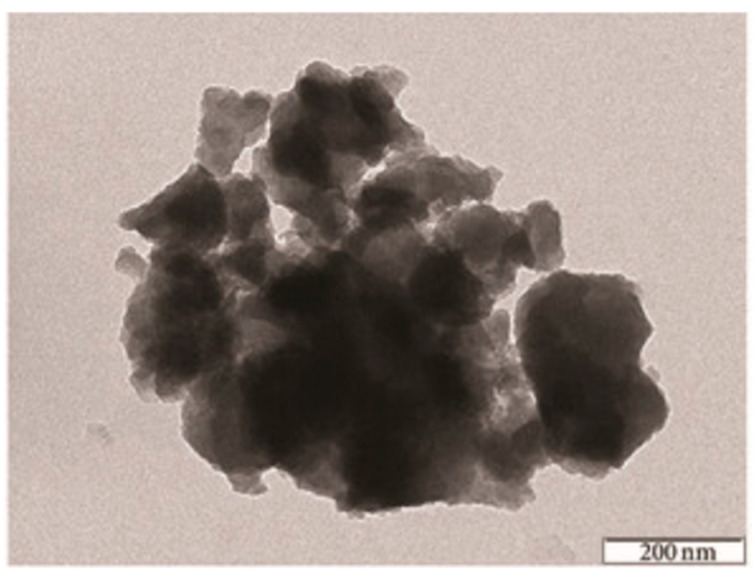
TEM analysis of an organic–inorganic hybrid synthesized by the sol–gel method, at a molar ratio of 2:1 (excess of phenyl phosphonic acid) [15].

The dye-sensitized solar cell (DSSC) is a low-cost solar cell from a group of thin film solar cells, based on a semiconductor formed between a photo-sensitized anode and an electrolyte. The surface structure and the morphology of such hybrids synthesized using the sol–gel methods, containing phosphorus and zirconium, starting from phenyl phosphonic acid and butyl-zirconate as precursors, could be proved and analyzed by SEM and TEM images (Figure 2 and Figure 3) [15].

**Figure 4:**
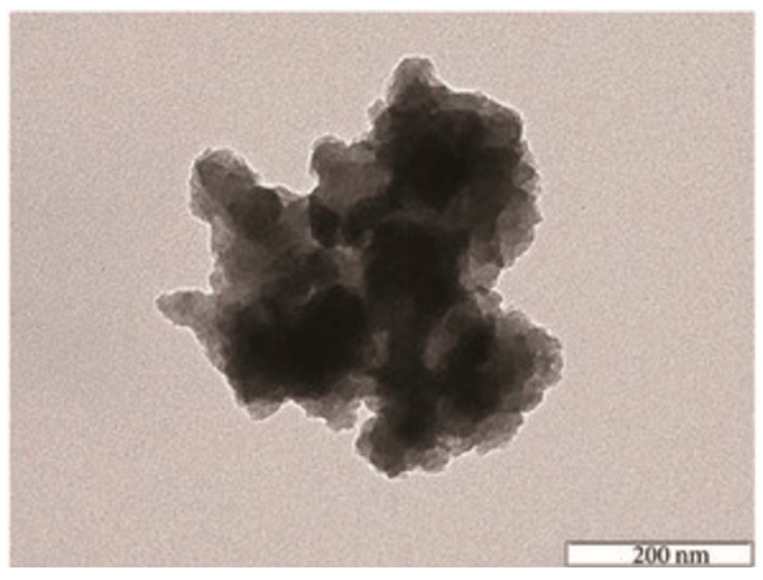
TEM analysis of an organic–inorganic hybrid synthesized by the sol–gel method, at a 1:1 molar ratio phenyl phosphonic acid: butyl-zirconate [15].

It can be observed that, as expected, the TEM analysis offers a better resolution of the obtained images than the SEM method. The TEM images (Figure 3 and Figure 4) also indicated porous structures of such hybrid materials. This porosity is required for DSSC applications. Therefore, by using the sol–gel process, it is possible to introduce a large variety of organic moieties into an inorganic matrix to obtain copolymer hybrid structures [6,8,9]. Metal phosphonate hybrids are one class of materials that could be obtained by using this method. For instance, zirconium phosphate of the class of tetravalent metal acid (TMA) salts was already investigated for proton transport properties [6]. In such hybrid materials, the proton transport properties of Zr(IV) phosphonate increased significantly in comparison with Zr(IV) phosphate itself [19,20].

**Figure 5:**
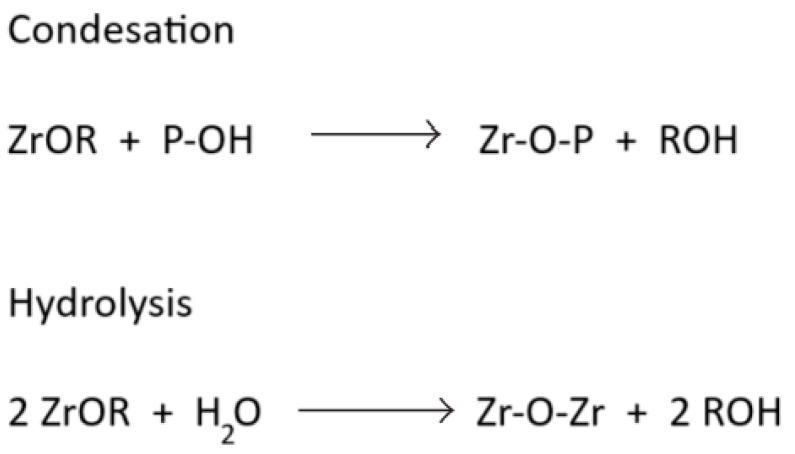
Schematic representation of the sol–gel process steps, of condensation and hydrolysis, if an alkyl-zirconate (ZrOR) is used as precursor.

If an alkyl-zirconate is used as a precursor, for a zirconium source, as in the examples from above, the chemical processes of the sol–gel steps will occur as follows and as shown in the schematic representation from Figure 5.

Organic–inorganic hybrid materials have the advantage of combining the properties of organic and inorganic moieties. The main goal of such materials is to control the influence of one part or the other. In some such hybrids, the ionocovalent bonds between the organic and the inorganic components are made via coupling agents. Some examples of coupling agents are organoalkoxysilanes [13,23], carboxylates, aminoalcohols [49], or phosphonic acids and their derivatives [50]. Alberti et al. synthesized alkyl and phenyl phosphonates containing zirconium, by using metal phosphonates [2,4,50].

In addition to their high thermal stability and low solubility, the porosity is also important. All of those properties of the products obtained by using the sol–gel method could be controlled. For example, it was proved that the P/M ratio has a significant influence on the porosity of the obtained products [14,16,24,25] because this ratio influences the chemical structure, the order of the repeating units, and nevertheless, the ratio/the order of P-O-M and M-O-M bridges. Their porosity could be changed by the used precursors, including the metal involved, and also by the ratio of those precursors in the sol–gel process. We observed all of these in our previous studies by SEM analysis [15].

In general, phosphinic and phosphonic acids, and also their derivatives, are used as precursors. Polyphosphonates can be also used as precursors [26,51,52]. In this case, even polymers could be obtained. For example, PEG could be used as a precursor together with zirconium compounds, in a sol–gel process as well. Those hybrid materials inhibit the different bacteria from growing. The same effect is obtained when silver is used in the structure of the precursors involved. Nevertheless, the phosphate-alumina coatings (starting from Al_2_O_3_ as a precursor) for obtaining FeSiAl magnetic composites, and also the photocatalytic reduction of graphene oxide nanosheets on TiO_2_ thin film for photoinactivation of bacteria in solar light irradiation, are the other two examples of the sol–gel method used to synthesize hybrids. The coating is a process used to obtain new composite materials, with supramolecular structures, including colloidal systems, such as thin films, for instance. Such films could behave as membranes and could interact with bacteria at the interfaces to inactivate them.

Aluminum–organophosphorus hybrid materials could be synthesized starting from aluminum oxide hydroxide (boehmite) and alkyl and aryl phosphoric acids as precursors. Such hybrids are further used to prepare composites with epoxy resin. This was proved to be a good solution to improve the flame retardancy of polymers. The epoxy composite containing 17 wt% of raw boehmite or hybrids was prepared to assess their flammability in the work of Dziuba et al. [53].

Following such green syntheses, all of the obtained materials, sol, gel, aerogel, xerogel, hydrogel, solid foam, and dry and dehydrated gel, are, in fact, colloids. For instance, a hydrogel is a biphasic material, containing a mixture of porous solids and at least 10% water. Sometimes, such gels could contain also other fluids, but in general, water is used. On the other hand, an xerogel is a network obtained after the removal of swelling agents from the gel. The sol–gel method could also be used to obtain xerogels. For a better understanding of all those types of gels, Table 1 describes the content of different colloidal systems.

The mentioned types of gels and/or gel-like materials could behave as chemical sensors and some of them could be used for the treatment of different types of cancer (lung, breast, and osteosarcoma) [27,54,55]. Most of the applications of such hybrids, also including composite membranes and thin films, are mainly related to the interface properties. The interface obtained after the sol–gel process could be controlled, as hydrophobic/hydrophilic, by using more polar groups or on the other hand, by using aromatic radicals and/or longer hydrocarbon chains in the structures of the precursors. The antibacterial effects of such composites, containing hybrids, are mainly related and influenced by the release of the metal (in general, hybrids containing silver have the best performances for such applications). The antibacterial coatings showed antibacterial activities of Ag–TiO_2_/Ag/a-TiO_2_ nanocomposite thin film photocatalysts under solar light irradiation. Moreover, different hydrogels (as previously described), biomimetic membranes, and hybrid materials were used in the fields of tissue engineering templates for the stimulation of human neural stem cells (for instance, on graphene/TiO_2_ heterojunction for differentiation into neurons). Graphene is a modern carbon material already used in some applications. It has a huge potential for more applications in different fields of great interest, while it is also a precursor for other carbon-based materials (i.e., nanotubes and fullerenes).

As for the mechanism of the sol–gel process, Vioux et al. [9,14,16,17,18] described a two-step sol–gel method used for the synthesis of ZrO_2_- and TiO_2_-phenylphosphonate hybrids as follows: the first step is the condensation of phosphonic/phosphinic acid or derivative with a metal alkoxide, obtaining M-O-P bridges, and then the second step is the hydrolysis–condensation reactions of the remaining metal alkoxide groups, obtaining M-O-M bridges [14,16,18] (where M represents the metal used).

In those organic–inorganic hybrids, the bridges between P atoms and M atoms, and also between two metal atoms (if there is an excess of the used precursor containing the metal), are always made via an oxygen atom. The M-O-P bridges formed during the condensation step of the sol–gel process, resulting in the phosphoryl oxygen coordination to metal atoms. Then, in the second step of the sol–gel process, the M-O-M bridges are obtained. It is not possible to obtain P-O-P bridges because the condensation of two P-O-H groups does not take place under the mild conditions involved in the sol–gel process. If the hybrid will be synthesized in the presence of boron-containing precursors, it is important to mention that boron will be in direct “competition” with phosphorus for metal ions (i.e., zirconium, titanium, and so on). In this case, during the first step of the sol–gel process, two types of bridges will be obtained: P-O-M and also B-O-M. This is also a function of the ratio, but this time, the P/B ratio is the most important. It should be taken into account that boron is not a metal and that the B-O-M is evidently not an M-O-M bridge. Boron, at the border between metals and non-metals, is very often called a metalloid. Practically, it is the only non-metal from its group, which further contains aluminum, for instance. The presence of boron could not lead to the obtainment of P-O-B bridges (as P-O-Zr or P-O-Ti bridges observed in the case of hybrids containing zirconium or titanium). Moreover, boron is just before carbon in the periodic table of the elements. As expected, boron has the ability to form stable covalent bonds in chemical molecular structures. It forms regular boron icosahedra. Boron is also a very hard material and it brings even more mechanical resistance to the obtained hybrid materials, in addition to the presence of zirconium or titanium.

And then, in the second step of the sol–gel process, M-O-M bridges will be obtained by hydrolysis. Anyway, in this case, in general, there will more likely be no metal ions available after the first step, because the metal will be involved in bridges with phosphorus, but also with boron, as shown. In other words, in this case of using three precursors (containing phosphorus, boron, and zirconium), the zirconium should be in excess, to form P-O-Zr and B-O-Zr bridges in the condensation step, but also have enough zirconium remain to also lead to the obtainment of Zr-O-Zr bridges in the hydrolysis step. Moreover, it is important to mention that no direct P-M, P-P, B-B, or M-M bonds were obtained [9,14,24]. Therefore, the chemical connection between phosphorus and zirconium, titanium, boron, and so on, or between metal atoms, is always made via an oxygen atom.

The sol–gel process can be performed in non-hydrolytic (non-aqueous) or in hydrolytic conditions. The non-hydrolytic sol–gel process [16,24,51] uses, as the main reaction, the condensation between a phosphonic acid (or a polyphosphonic acid) and a metal-containing compound (for instance an alkoxide) [17]. There are two main parameters that have a significant influence on the structures of the obtained phosphorus-containing hybrid materials: the P/M ratio [14,16,24,25] and the nature of the precursor containing phosphorus used as coupling agents. For instance, tetravalent metal phosphonates (M = Ti, Zr, and so on) usually feature [RPO_3/2_]^2−^ tetrahedra and [MO_6/2_]^4+^ octahedra connected through M-O-P bridges. If the phosphorus-containing precursor is in excess, more P-O-M bridges are expected to be obtained in the first step. At the same time, almost no metal ions will remain to form M-O-M bridges in the second step of the sol–gel synthesis. On the other hand, if the precursor containing the metal is in excess, after the first step of condensation, the reaction mixture will still retain enough metal ions to also lead to the significant formation of M-O-M bridges in the second step of hydrolysis. Therefore, the structure and also the porosity of the obtained hybrid materials are strongly influenced by the molar ratio used.

It was observed in the case of organic–inorganic hybrids containing phosphorus and zirconium that they are not soluble and have high thermal stability. Once the sol–gel synthesis is complete, the insoluble product lead to the obtainment of sediment. The sediment is then separated by decantation, and subsequently washed and filtered several times with water. After all of those procedures, the final step of product purification is drying in an oven for a few hours at 60–80 °C. After drying, the obtained material became a porous dry gel. The pore’s size and their distribution could be changed and controlled by the synthesis parameters (i.e., the molar ratio of the precursors, the use of additives, surfactants, and so on). The dry gel, sometimes so-called “solid foam”, is also a colloidal system, such as a sol–gel.

The structure of the hybrid materials containing phosphorus compounds can be studied by several spectroscopic methods that could analyze solid materials, such as FTIR [16,28], X-ray photoelectron spectroscopies [29], SEM, TEM, TGA, EDX, and so on. These analysis methods can prove the presence of M-O-P and M-O-M bridges, as well as the presence of residual M-O-H, P-OH, and P=O bonds. It should be pointed out that almost always, the structure of organic–inorganic hybrids containing phosphorus synthesized using the sol–gel method will also retain such residual chemical bonds (M-O-H, P-OH, and P=O) that did not react completely.

The microscopic images, either SEM or TEM, show the morphology of the hybrid material surface. In general, for such materials, compact structures of 50–200 μm are observed. The EDX results are used to confirm the chemical elements from the hybrid structure. The compact morphology of the hybrid compounds obtained by using sol–gel process is shown by SEM and/or TEM images. On the other hand, it should mentioned that the EDX method offers qualitative and quantitative analytical information. In general, in those organic–inorganic hybrids, it is expected to have a relatively high amount of oxygen, because oxygen is involved in both the P-O-M and M-O-M bridges (Table 2), and also in the residual P-OH and P=O bonds, as already mentioned.

When analyzed by TGA, high thermal stability was proved for those hybrid materials synthesized by the sol–gel method (the mass loss was in general below 30% at 900 °C, when the analysis was performed in air and also in nitrogen). Some hybrids did not even show a mass loss at all from 700 to 900 °C; therefore, the mass loss at 700 °C was at the same time as the maximum mass loss, in the temperature range studied (25 °C–900 °C). Those properties make them interesting materials also for applications at high temperatures.

If we compare those organic–inorganic hybrids containing phosphorus and zirconium with other polymers containing phosphorus, both synthesized by green methods, the properties could be similar, but not always. In Table 3, such similar properties and also some possible differences in the obtained compounds are mentioned.

It can be observed that the organic–inorganic hybrids containing phosphorus have good thermal stability and are generally not soluble. On the other hand, the polymers containing phosphorus, synthesized by using UV curing, are soluble in general, and at the same time, are not very stable from the thermal point of view. The sol–gel synthesis of hybrid phosphorus-containing materials involves the reaction of metal or silicon precursors with phosphonate “coupling molecules” (usually phosphonic acids, biphosphonic acids, polyphosphonic acids, and their derivatives, or even phosphonate sodium or ammonium salts in aqueous or in anhydrous conditions) with or without a templating agent [16]. The templating agent is especially used to control the pore size and the material porosity. This is another option to control and change the material porosity, in addition to the precursors used and their ratio. On the other hand, if there is no template, the porosity results only from the voids remaining after the solvent evaporation, during the drying process (in order to obtain the dry foam). It is necessary to have a high degree of condensation to avoid the pore collapse [28,29,30,31,32,56,57].

### 2.3. The Use of Surfactants in the Sol–Gel Process

The applications of organic–inorganic hybrid phosphorus-containing materials are related also to their specific surface area. Small amounts of organic additives (for example, different copolymers containing ethyleneoxide groups such as PEG, nonionic surfactants, and so on) could influence the sol–gel process when added. The additives [56] will not directly influence the chemical structure, but the physical properties and the supramolecular structure of the synthesized hybrids could be affected. For example, the surfactants will influence the porosity and the surface area of the obtained materials.

The surfactants are molecules containing a polar group and a hydrophobic tail (in general, a long hydrocarbon chain). If surfactants are added, more forces will appear during the sol–gel process, the function of the charge, surface tension, surfactant concentration, hydrophobic/hydrophilic properties, and so on, in agreement with the DLVO theory of colloidal systems [58,59,60,61,62]. Those forces could influence the sol–gel process from a kinetics point of view. As a consequence, the surfactants could also make the sol–gel process faster. If an anionic or a cationic surfactant is added to the sol–gel synthesis, their charge could lead to aggregation and this will not help the process. But if a non-ionic surfactant is added, the aggregation will not appear anymore.

Some of the most important surfactants that influenced the sol–gel syntheses are alcohols with a long hydrocarbon chain. Starting from butanol, such alcohols with hydrophobic hydrocarbon chains showed significant surface activity. Those alcohols are non-ionic surfactants. If alcohols such as methanol or ethanol are used in the sol–gel process instead of water, or even in mixtures with water, they will not significantly influence the syntheses, because they are very polar molecules. But, if alcohols with long hydrocarbon chains, with higher hydrophobic properties, will be used, they will behave as non-ionic surfactants. In comparison with classical non-ionic surfactants, which also contain ethylene-oxide groups (CH_2_CH_2_O), those alcohols have no critical micellar concentration (cmc). Therefore, the hydrophobic alcohols do not form micelles. Instead, they will phase separately at a certain ratio, when used in mixtures with water. Therefore, an interface will appear, and this will influence the kinetics of the sol–gel process. Alcohols from butanol to octanol behave as described here. If alcohols with an even higher hydrophobic carbon chain are involved (i.e., decanol, dodecanol, and so on) or classical non-ionic surfactants with ethylene-oxide groups, the mixture will lead to obtaining a very stable foam. This foam will completely stop the sol–gel syntheses. As a consequence, it can be concluded that only alcohols from butanol to octanol influenced the sol–gel process by changing its kinetics, due to their surface properties. The explanation for this is relatively simple: those alcohols, instead of forming micelles, will phase separately at a certain concentration (for instance, the ratio 1:1 vol. water: alcohol was high enough to obtain the separation interface). If the interface was more stable, the sol–gel process was faster. But, only until a stable foam appeared did the syntheses actually stop, as previously mentioned.

The obtained interface was more stable if the hydrocarbon chain from the alcohol’s structure was longer (more hydrophobic). This is due to the forces, which will appear in the reaction mixture, in agreement with the DLVO theory. In this case, the most important will be the hydrophobic interactions. Those hydrophobic forces are always attractive and as a consequence, the organic part from phosphorus-containing precursors (for example, phenyl groups from phenyl phosphonic acid, phenyl phosphinic acid, and so on) will be present at the interface. They are attracted at the interface by the hydrophobic forces generated by the long hydrocarbon chain from the alcohol’s structure (used as a surfactant). In this way, the groups involved directly in the synthesis will be free to react and finally lead to the obtainment of P-O-M bridges. For those reasons, the sol–gel process will be faster when such alcohols with surfactant properties are used.

If the added surfactant would have a charge, it would therefore be either anionic or cationic, and an aggregation would occur between the hybrid and the surfactant molecules. Such systems could be compared with mixtures containing surfactants and polyelectrolytes with opposite charges, where significant aggregation occurs at the so-called critical aggregation concentration. In the case of the sol–gel process, the aggregation will slow the synthesis and it could also reduce the yield of it.

Therefore, if surfactants are used in a sol–gel synthesis, alcohol with a long hydrocarbon chain (from butanol to octanol) will make the process faster due to the stable interface formed when the phase separation occurred and due to the hydrophobic forces. If the alcohol is too polar (i.e., methanol, ethanol), it will be also very soluble in the aqueous phase and as a consequence, no interface will appear (no influence on the sol–gel synthesis). It is good to have a non-polar alcohol, in order to obtain a phase separation and also a stable interface. Those alcohols are still soluble in water at low concentrations but will phase separate at high concentrations.

On the other hand, if the hydrocarbon chain from the surfactant structure is too long (and as a consequence too hydrophobic), the alcohol will produce a stable foam when added, and the foam will stop the sol–gel synthesis (no hybrid product will be obtained in this case).

## 3. Conclusions

The sol–gel process is a green synthesis used for obtaining organic–inorganic hybrid compounds such as copolymer networks containing phosphorus, zirconium, titanium, and boron, starting from different precursors such as phenyl phosphonic acid, phenyl phosphinic acid, polyphosphonates, and their derivatives, by using mild conditions. The sol–gel syntheses take place at room temperature by using water or alcohol as solvents. The obtained hybrids are very stable from a thermal point of view. In comparison with classical syntheses, the organic–inorganic hybrid materials synthesized by the green sol–gel method showed higher thermal stability. Therefore, their thermal properties have been improved significantly when the sol–gel method was involved. Hybrid materials are very useful for different applications of great interest nowadays as sensors, water electrolysis units, DSSC [63], and other electrochemical devices, and also in the fields of catalysis and medicine [24,25,26,27,28,29,30,31,32,33,34,35,36,37,38,39,40,41,42,43,44,45,46,47,48,57]. Green sol–gel syntheses could lead also to obtaining magnesium oxide nanoparticles and different nanocomposites for photocatalytic antimicrobial, antibiofilm, and antifungal applications [64]. The metal–organic frameworks (MOFs) showed, in general, the potential for biomedical applications [65]. There are also hybrids containing phosphorus, zirconium, and boron. In this case, boron will be in direct competition with phosphorus in the first step of the sol–gel process to lead to the obtainment of Zr-O-B bridges. Small amounts of boron will give even stronger mechanical resistance to the obtained materials. But, for applications in medicine, it should be always taken into account if those materials are biocompatible. The applications in medicine are mainly related to biocompatible materials obtained by the sol–gel synthesis, solid materials used to replace different tissues, such as compact bone tissues, and gels or gel-like materials used in several therapies for different types of cancer. The strong mechanical properties of such hybrid materials are necessary for obtaining prostheses to replace compact bone tissues. Depending on the used precursors, the hybrid materials synthesized by using the sol–gel method, could have stronger mechanical resistance (for instance, the metal–organic frameworks containing zirconium or titanium). A large variety of organic moieties could be introduced into an inorganic matrix by the sol–gel synthesis in order to obtain copolymer hybrid structures, the function of the application of the obtained hybrid networks.

## 4. Outlook

In the future, we would like to use the sol–gel process for obtaining novel organic–inorganic hybrids starting from different precursors containing phosphorus, zirconium, and boron, even by using precursors with organic aliphatic radicals in their structures, not only aromatic as in most of the cases described in the present manuscript. And, nevertheless, we would like to find more applications for those materials. Our recently published article [27] describes and confirms the applications of some organic–inorganic hybrids (starting from phosphonic and phosphonic derivatives) for the treatments of different human osteosarcoma. We plan to continue the research in this direction, of course also connected to other applications such as the ones mentioned above.

## Figures and Tables

**Table 1:** Different types of colloidal systems.

Dispersion Medium	Dispersed Phase		
	Gas	Liquid	Solid
Gas	-	Liquid aerosol (fog, clouds, steam, spray)	Solid aerosol (smoke, ice clouds)
Liquid	Foam	Emulsion, liquid crystals	Sol, sediment, precipitates, suspension, aggregates
Solid	Solid foam, aerogel	Gel, hydrogel, gelatine, jelly	Solid sol

**Table 2:** The possible and not possible chemical bridges that could be/could not be obtained on the structure of the hybrids synthesized by the sol–gel syntheses when precursors containing phosphorus, zirconium, and boron are involved.

Bridges	Sol–Gel Step	Chemical Reaction
Zr–O–P	During the first step	Condensation
Zr–O–Zr	During the second step	Hydrolysis
Zr–O–B	During the first step	Condensation
P–O–P	Not possible in mild conditions	
B–O–B	Not possible in mild conditions	
P–O–B	Not possible in mild conditions	

**Table 3:** The comparison of different properties of hybrids/polymers containing phosphorus synthesized by different green methods.

Sol–Gel	Surface Grafting	Polymerization by UV Curing
Not soluble	Not soluble (the synthesis could be conducted even in solid state)	Generally soluble
Mass loss up of 20–30% at 500 °C	Mass loss up to 50% at 500 °C	Mass loss around 60% at 500 °C
Mass loss up to 40% at 700 °C	Mass loss up to 60% at 700 °C	Mass loss up to 70% at 700 °C
No further mass loss observed above 700 °C until 900 °C	Mass loss still slowly increase until 900 °C	Mass increase significantly above 700 °C

## Data Availability

The data presented in this study are available from the corresponding author upon request.
